# Magnitude of paternal postpartum psychological distress and associated factors in Addis Ababa, Ethiopia: a facility-based cross-sectional study

**DOI:** 10.1186/s12888-023-04891-w

**Published:** 2023-06-01

**Authors:** Addisu Tuji, Subah Abderehim Yesuf, Ribka Birhanu, Barkot Milkias

**Affiliations:** 1 Department of Psychiatry, Tirunesh Beijing General Hospital, Addis Ababa, Ethiopia; 2 Department of Family Medicine, St. Peter’s Specialized Hospital, Addis Ababa, Ethiopia; 3 Department of Psychiatry, School of Medicine, College of Health Sciences, Addis Ababa University, Addis Ababa, Ethiopia

**Keywords:** Paternal postpartum psychological distress, Associated factors, Kessler-10, Ethiopia

## Abstract

**Background:**

The psychological distress of fathers in the postpartum period can have adverse effects on the well-being of the family and the newborn’s development in particular. However, fathers’ mental health throughout the postpartum has remained understudied and clinically overlooked in many developing countries, including Ethiopia. This study aims to assess the prevalence of psychological distress among fathers in the postpartum period and to examine the associated factors in an Ethiopian population.

**Methods:**

A facility-based, cross-sectional study was conducted at Tikur Anbessa Specialized Hospital (TASH) and Gandhi Memorial Hospital (GMH) in Addis Ababa, Ethiopia. A systematic sampling method was employed to include 280 fathers whose partners gave birth 6 to 8 weeks before the interview. Psychological distress was assessed using a validated Amharic version of the Kessler Psychological Distress Scale (K10) through a telephone interview. The collected data was analyzed using SPSS version 26. Descriptive statistics were used to summarize the data. Multivariable logistic regression was run to determine the variables associated with paternal postpartum psychological distress (K10 total score ≥ 7, a validated cut-off score in an urban Ethiopian setting), and odds ratio with 95% confidence intervals were obtained. A two-tailed p-value < 0.05 was considered for statistical significance.

**Results:**

About one-fifth of the fathers endorsed having distress symptoms during the postpartum period. Those with lower income (AOR = 11.31, 95% CI:  4.10, 31.15), unintended pregnancy (AOR = 3.96, 95% CI: 1.02, 15.46), poor social support (AOR =3.28 95% CI: 1.43, 7.50), poor infantile health (AOR = 8.20, 95% CI: 2.35, 28.66)  and maternal postpartum distress (AOR = 12.10,  95% CI: 3.15, 46.48) had significantly higher odds of having paternal postpartum distress.

**Conclusions:**

Paternal postpartum distress was present in one-fifth of the fathers included in this study. This calls for due attention and efforts for early detection of those at risk of paternal distress and the development of interventions that consider their specific needs.

## Introduction

The birth of a baby and transition to parenthood can be an exciting and unique life experience that significantly impacts parents’ lives. Yet, both parents also have to face the biological and psychosocial changes accompanying the postpartum period. For fathers, this period can be a chapter of great upheaval and anxiety that can perturb the status of their relationship and the life of the newcomer [1].

Psychological distress is a mental state characterized by disturbance in emotion regulation; it manifests with non-specific symptoms such as depression, anxiety or somatic dysfunction [2]. Studies in various settings have shown that higher levels of psychological distress are predictive of common mental disorders (CMDs) and significant functional impairment [3, 4].

It is well established that postpartum is a particularly vulnerable period for the onset of depressive and anxiety symptoms in mothers [5, 6]. Most of the literature on the postpartum period is focused on the prevalence, risk factors and interventions for maternal CMDs. Although psychological disturbances in either of the parents can affect the well-being of the other family members [7], paternal distress has not received much research and clinical attention [8, 9].

The mounting evidence on paternal postpartum psychological disturbances indicates that the burden of mental distress in fathers is as significant as that of the mothers, in some studies [8, 10–12], with nearly similar incident rates of depression [13]. Estimates of the prevalence rates of paternal perinatal emotional disturbance, including stress, anxiety and depression, range from 1.2% in Ireland, 5.2% in China,16.6% in Saudi Arabia to 59.8% in Iran [14–17]. Apart from the varied study settings and research methods, this wide variation can be partly explained by the lack of consensus on case definition, validated screening tools and cut-off points, given the relative novelty of the area.

Fathers experiencing psychological distress suffer from many emotional and physical disturbances that compromise their quality of life and level of functioning, leading to a more significant impact on the family. Reported adverse outcomes on the family include an increased risk of maternal depression [18], increased conflict in the marital relationship and violence against mothers [19], impaired father–infant relationship, poor infant emotional, cognitive and social development, and psychiatric morbidity that may extend into late adolescence [19–24]. In addition, paternal psychological distress influenced the quality and quantity of fathers’ involvement in childcare [25].

Steps toward alleviating the burden of paternal postpartum psychological distress require understanding the risk factors and the buffering factors. Several sociodemographic, psychosocial, behavioral and pregnancy and delivery related factors were identified to escalate the development of psychological distress among fathers across different contexts [15, 17, 26]. However, these associations remain unclear and inconsistent [10, 27], particularly from the Ethiopian perspective.

The scarce studies on paternal postpartum mental health have limited focus on postpartum depression and are predominantly from high-income countries (HICs) [28]. There remains a dearth of evidence regarding the psychological stress among fathers from developing countries, leaving a gap in knowledge that needs to be addressed. Thus, with such a notion, this study was conducted to explore the magnitude of postpartum psychological distress and the associated factors among fathers whose partners delivered in two referral hospitals in Ethiopia.

## Methods

### Study setting and participants

The study was conducted in two hospitals located in Addis Ababa, Ethiopia. The participants were recruited at Gandhi Memorial Hospital and Tikur Anbessa Specialized Hospital from June 15 to September 15, 2020. These hospitals are among the largest and oldest public hospitals in the country, providing a high level of clinical care, including maternity care for over eight million people. In addition to providing routine antenatal care, the obstetric units of both hospitals are among the top referral hospitals for high-risk pregnancies and deliveries.

Although the first year after childbirth is regarded as the postpartum period, the focus of the study will be the occurrence of symptoms in the critical first two months postdelivery. Eligibility criteria for included fathers were having partners who gave birth at either of the hospitals six to eight weeks before the time of the interview period, being able to communicate in Amharic language, being able to consent to participate in the study, and having a registered phone number in the medical record of their partners. Fathers who were too acutely disturbed to engage in an interview, fathers who self- reported getting divorced or widowed between the time of childbirth and data collection and fathers with a severe communication disorder were excluded.

### Tools and data collection

Psychological distress was measured using the Kessler 10-item Psychological Distress Scale (K10), which has been extensively used and validated in Ethiopian settings. The tool assesses the presence of general distress by inquiring about symptoms experienced in the past month. Each item is rated on a 5-point Likert scale from 0 ‘none of the time’ to 4 ‘all of the time’, with the total score ranging from 0 to 40. The Amharic version has demonstrated good psychometric properties in urban and rural Ethiopian settings [3, 29, 30]. A validation study in Addis Ababa on postpartum women has shown good internal consistency (Cronbach’s α = 0.90) and generated sensitivity and specificity of 84.2% and 77.8%, respectively, at optimal cut-off point ≥ 7 [29]. Thus, in this study, paternal postpartum psychological distress was defined as psychological distress measured by K-10 (total score ≥ 7) in a period that spans from the delivery of an offspring to six completed weeks afterward.

Because of the need to exercise the WHO-approved precautionary measures of physical distancing to prevent COVID-19 transmission, telephone-based interviews were conducted by two trained professional nurses. The data was collected under close supervision and facilitation by the principal investigator.

### Sample size determination

The single population proportion formula was used to calculate the sample size with the assumption of 5% margin of error, 95% confidence interval and 22.8% as proportion of fathers with postpartum depression from a previous study [25] and adding a non-response rate of 10%. Accordingly, the required sample size was 287.

### Sampling procedure

The total number of mothers who gave birth starting from the previous six months till the time of data collection was reviewed from the maternity wards of each study hospital, where the delivery registry is available, to compute the average number of deliveries per month. Accordingly, both hospitals had an approximate average of 750 deliveries per month. As the study was designed to be conducted over three months, a total of 2250 deliveries were estimated for each hospital; hence, using a systematic sampling method, every eighth mother’s partner (K = 2250/287 = 7.8≈8) was approached for participation. The first samples in each hospital were chosen by a lottery method.

### Statistical analysis

The collected data were entered, cleaned and analyzed using SPSS version 26. Descriptive statistics were used to summarize the sociodemographic data. Binary and multiple logistic regression analysis was used to ascertain the association between the outcome and explanatory variables. Hosmer-Lemeshow goodness-of-fit was used to test model fitness. The odds ratio and the 95% CI were computed to quantify the association between covariates and the outcome variable. Covariates with a p-value of ≤ 0.2 on the bivariable analysis were included in the multiple logistic regression to control all possible confounding factors. For all statistical tests, a two-tailed p-value of ≤ 0.05 was used as a cut-off point for statistical significance.

## Results

### Background characteristics of study participants

A total of 287 fathers were recruited in this study within 6 to 8 weeks after their partners gave birth. Two hundred and eighty fathers were successfully interviewed, making a response rate of 94.3%. The participants’ age ranged from 23 to 50 years, with a median (interquartile range) of 31 [28–38] years. Of all participants, 40% (n = 112) were aged from 23 to 29 years, and 37.1% (n = 104) reported having only completed primary school. Nearly 39% (n = 109) were merchants, while 42% (n = 118) reported having an average monthly income ranging from 5001 to 10,000 ETB (Table 1).

**Table 1:** Background characteristics of fathers whose partners gave birth in GMH and TASH, AA, Ethiopia, 2020

Paternal characteristics	Frequency N = 280	Percent (%)
**Age category (years)**		
23–29	112	40.0
30–34	68	24.3
35–39	36	12.9
40–44	36	12.9
45–50	28	10.0
**Educational status**		
Uneducated or primary school	111	39.6
Secondary school	95	33.9
College diploma+	74	26.4
**Occupation**		
Merchant	109	38.9
Employee	103	36.8
Daily laborer or unemployed	68	24.3
**Average monthly income**		
< 1000 ETB	16	5.7
1000–5000 ETB	94	33.6
5001–10,000 ETB	118	42.1
> 10,000 ETB	52	18.6
**Number of household children**		
1	126	45.0
2	91	32.5
3	47	16.8
≥ 4	16	5.7
**Pregnancy intention**		
Unplanned	16	5.7
Planned	264	94.3
**Infant sex**		
Male	124	44.3
Female	156	55.7
**Infant health**		
Good	247	88.2
Poor	33	11.8

GMH: Gandhi Memorial Hospital; TASH: Tikur Anbessa Specialized Hospital; AA: Addis Ababa

Almost half of the participants (45%) reported having only one child at the time of data collection. Most (94.3%) described that the pregnancy was planned, and over half (55.7%) of the newborns were females. The general medical condition of the newborns was described as good in 88.2% of the cases (Table 1).

### Psychosocial characteristics of participants

Most (92.5%) of the participants stated not to have any significant conflict between their work and family life, while the majority (82.9%) claimed to be satisfied with their marital life. One hundred seventy-one (61.1%) participants reported having social support. Almost all (99.3%) of the fathers claimed not to have any chronic medical comorbidity. In contrast, only 3.9% endorsed having been previously diagnosed with a certain mental disorder by a health professional. When asked about their partners’ perinatal mental well-being, 8.6% noted features of psychological distress during the index peripartum period, and almost all (98.9%) of the fathers who participated in this study stated not to have any family history of psychiatric illness (Table 2).

**Table 2:** Psychosocial characteristics of fathers whose partners gave birth in GMH and TASH, AA, Ethiopia, 2020

Variable	Frequency N = 280	Percent (%)
**Work-family conflict**		
Yes	21	7.5
No	259	92.5
**Marital satisfaction**		
Yes	232	82.9
No	48	17.1
**Social support**		
Yes	171	61.1
No	109	38.9
**Medical illness**		
Yes	2	0.7
No	278	99.3
**Mental illness**		
Yes	11	3.9
No	269	96.1
**Maternal distress**		
Yes	24	8.6
No	256	91.4
**Family history of distress**		
Yes	3	1.1
No	277	98.9

GMH: Gandhi Memorial Hospital; TASH: Tikur Anbessa Specialized Hospital; AA: Addis Ababa

### Magnitude of paternal postpartum distress

Overall, the prevalence of paternal psychological distress within 6 to 8 weeks postpartum was 19.3% (n = 54).

### Factors associated with psychological distress

In this study, fathers with an average monthly income of not more than 5000 ETB had higher odds (AOR = 11.31,  95% CI: 4.10, 31.15) of experiencing distress postpartally than those who earn more than 5,000 ETB a month. Similarly, fathers who reported that the index pregnancy was unintended had 3.33 times more odds of having psychological distress compared with those who planned the pregnancy (AOR = 3.96, 95% CI: 1.02, 15.46) (Table 3).

In comparison to fathers whose babies were in good health condition, those who reported otherwise had more odds of having psychological distress (AOR = 8.20, 95%CI: 2.35, 28.66). Fathers who claimed to have lacked social support had more than three-fold increased odds of experiencing postpartum paternal distress (AOR = 3.28, 95% CI: (1.43, 7.50) in reference to those who had some form of social support. Moreover, fathers whose partners had features of postpartum distress had statistically higher odds of having psychological distress when compared to those that did not report maternal distress (AOR = 12.10, 95% CI: (3.15, 46.48) (Table 3).

**Table 3:** Binary and multiple logistic regression model showing factors associated with postpartum psychological distress among fathers

Variable*		COR (95% CI)	P value	AOR (95% CI)	P value	
Educational Status	Uneducated or Primary school	2.40 (1.02, 5.66)	0.046	1.43 (0.38, 5.46)	0.600	
	Secondary school	2.34 (0.97, 5.64)	0.060	0.59 (0.16, 2.19)	0.430	
	College diploma+	1 (Reference)	-	1 (Reference)	-	
Occupation	Merchant	1 (Reference)	-	1 (Reference)	-	
	Employee	0.99 (0.46, 2.12)	0.980	1.68 (0.47, 5.92)	0.420	
	Daily laborer or unemployed	2.97 (1.43, 6.17)	0.003	2.17 (0.77, 6.09)	0.140	
Income	≤ 5000 ETB	10.67 (5.07, 22.45)	< 0.001	11.31 (4.10, 31.15)	< 0.001	
	> 5000 ETB	1 (Reference)	-	1 (Reference)	**-**	
Pregnancy intention	Unplanned	8.30 (2.88, 24.12)	-	3.96 (1.02, 15.46)	0.048	
	Planned	1 (Reference)	< 0.001	1 (Reference)	**-**	
Infant’s health	Good	1 (Reference)	-	1 (Reference)	**-**	
	Poor	5.18 (2.41, 11.13)	< 0.001	8.20 (2.35, 28.66)	0.001	
Marital satisfaction	Yes	1 (Reference)	-	1 (Reference)	**-**	
	No	4.16 (2.11, 8.21)	< 0.001	2.12 (0.80, 5.57)	0.130	
Social support	Yes	1 (Reference)	-	1 (Reference)	-	
	No	5.79 (3.00, 11.20)	< 0.001	3.28 (1.43, 7.50)	0.005	
Maternal distress	Yes	14.38 (5.58, 37.04)	< 0.001	12.10 (3.15, 46.48)	< 0.001	
	No	1 (Reference)	-	1 (Reference)	-	
Family history	Yes	8.65 (0.77, 97.25)	0.080	1.34 (0.30, 59.16)	0.880	
	No	1 (Reference)	-	1 (Reference)	-	

*Only variables with p-value < 0.2 in binary logistic regression are shown here; COR: Crude Odds Ratio; AOR: Adjusted Odds Ratio

## Discussion

To the best of our knowledge, this is the first Ethiopian study that tried to provide prevalence estimates and insights into the potential predictors of paternal postpartum psychological distress, an overlooked area of health care and research. In this study, we demonstrated that one-fifth (19.3%) of the studied fathers had psychological distress, indicating that a considerable proportion of fathers suffer from paternal postnatal distress. This is a crucial finding as its negative effects on the infant, the couple and the family are immense [19, 21, 22].

Our finding is congruent with the Korean and Iranian reports, where the corresponding figures for postpartum psychological distress were 22.8% [25] and 21% [31], respectively. It was comparatively lower than the magnitude observed among Mexican (29.5%) [32] and Malaysian studies [33], which detected psychological distress in 29.5% and 36.3% of fathers. Further, our finding was higher than the prevalence rates (13%) recorded among Norwegian fathers (13%) a few days after childbirth [11]. In contrast, our prevalence estimate was higher than the report of Giallo and colleagues, who noted that psychological distress during the postnatal period affected 9.7% of Australian fathers [34].

The discrepancies can result from the differences in the assessment tools used and cut-off points, the sample size, the constitution of the population studied and the timing of the study in relation to the timing of childbirth. With regards to measurement tools, we used Kessler-10 tool, while the Korean and Australian studies assessed parental psychological distress using the Kessler 6-item Psychological Distress Scale [25, 34]; the Iranian and Norwegian studies used a General Health Questionnaire-28 (GHQ-28) [11, 31] while the Malaysian study used the Depression Anxiety Stress Scale 21 to assess distress [33]. It is also worth noting that this study was done in the early months of the COVID-19 pandemic, which was implicated in causing serious psychological problems in various populations, fathers included [35–37]. Another critical point is that distress manifestations can have cultural variations, resulting in statistical observation fluctuations [38].

Furthermore, in keeping with previous studies [26, 32, 39], our study demonstrated that fathers with financial stress are at heightened risk of psychological distress. This can result from the fact that parenthood typically increases the need for fulfilling the materials and facilities needed for the family, including the baby’s basic consumption. Consequently, this economic anxiety might further affect the mental well-being of fathers during such a period.

Also, we found that unintended pregnancy was independently associated with psychological distress, and this corroborates with the observation of other authors [40, 41]. This can be attributed to various factors relating to socioeconomic position, such as the increased financial pressures of a new child, and psychological readiness for parenthood [41]. On the other hand, the finding that spousal psychological distress is a significant factor for paternal distress is unsurprising, as maternal depression is among the most commonly cited predictor of paternal distress [28, 42]. These studies have shown that maternal and paternal psychological well-being are interwoven. A mother’s depression is related to other risk factors for paternal distress directly or indirectly. It would affect the quality of the marital relationship, the support provided for the father and the health of the newborn. In the Ethiopian culture, where childcare responsibilities almost entirely fall on the shoulder of the mother, maternal depression compromises childcare and creates a more stressful postpartum experience for the father [43].

Furthermore, fathers who lacked social support were more likely to have psychological distress than those with support. This result was in agreement with a similar study conducted in Ireland and Hong Kong [14, 15] and a systematic review and meta-analysis [28]. This might be because poor social support can lead to a feeling of loneliness, which in turn may result in disturbances in mental well-being. Again, this might have been accentuated by the COVID-19 crisis as some authors have underscored the importance of communal postpartum rituals in the Ethiopian culture in relation to childbirth [44].

Finally, this study found that fathers whose children have poor general health are more likely to be distressed than those with a generally healthy child. Although a meta-analysis by Wang et al. [28] reported that no statistically significant association with infant factors was found, it also shed light that there is a lack of evidence on some infant factors. Our finding can be explained by the fact that compromised infant health can result in a wide range of issues, including emotional trauma, increased childcare burden and medical costs in the face of resource challenges, among others.

Our study has certain limitations to be considered when interpreting the results. It was a cross-sectional study, and thus, inferences about causality cannot be made. And because data collection was done through telephone interviews, possibly when the fathers were with their partners, there could be social desirability bias leading to underreporting of symptoms or factors associated. As the study sites selected were tertiary hospitals that serve as referral centers for high-risk pregnancies, our sample may not represent fathers that can be generalized to all fathers. Certain independent variables, such as social support and maternal distress, were entirely dependent on fathers’ subjective responses, and standardized measurements were not employed; these should be interpreted with caution. We also acknowledge that the COVID-19 crisis might have influenced the prevalence of mental distress as it had a multifaceted psychosocial impact.

## Conclusion and recommendations

Our findings indicate that postpartum psychological distress affects a significant number of fathers, demonstrating that it represents a public health concern. Paternal factors such as economic problems, lack of social support, compromised infantile health and partner’s distress are significantly associated with paternal postnatal distress. These factors highlight the need for early detection and screening for psychological distress in both parents. Health care providers should be made aware of these risk factors so that they will be able to educate, prepare and screen for mental health challenges, especially those at higher risk. Strengthening psychosocial support for fathers and tailoring psychiatric care to their needs may help fathers cope better with stressful experiences accompanying the birth of a child. Finally, this study lays the groundwork for further exploration of paternal mental health issues in the Ethiopian setting.

## Data Availability

The datasets used during the current study are available from the corresponding author on reasonable request.

## Abbreviations

CMDs: Common Mental Disorders
COVID-19: Corona Virus Disease-19
GMH: Gandhi Memorial Hospital
K10: Kessler Psychological Distress Scale
TASH: Tikur Anbessa Specialized Hospital
WHO: World Health Organization
